# New Medical College

**Published:** 1861-06

**Authors:** 


### New Medical College.

We are in receipt of the first Annual Announcement of the Medical College of Texas, to open its first session in Houston, Texas, on the third Monday in November next. The College is organized with the following Faculty:

Ashbel Smith, M. D., Prof. of Surgery.

N. N. Allen, M. D., Prof. of Anatomy.

G. A. Feris, M. D., Prof. of Theory and Practice Medicine.

W. H. Gautt, M. D., Prof. of Obstetrics.

W. S. Rodgers, M. D., Prof. of Diseases of Women and Children.

J. F. Matchet, M. D., Prof. of Mat. Med. and Therapeutics.

W. P. Riddell, M. D., Prof. of Chemistry and Med. Jurisprudence.

Thos. E. Brooks, M. D., Prof. of Physiology and Pathology.

B. P. January, M. D., Demonstrator of Anatomy.

